# Supplementary material for: Exome-wide association study reveals 7 functional variants associated with ex-vivo drug response in acute myeloid leukemia patients
Source: BMC Med Genomics. 2025 Apr 4;18:64. doi: 10.1186/s12920-025-02130-7 (PMC11969768; doi:10.1186/s12920-025-02130-7)
Supplement: Supplementary file 1 — Supplementary Material 1 [file 12920_2025_2130_MOESM1_ESM.zip › Supplementary_material/Supplementary Information.docx]

**Supplementary Information**

**Supplementary Figure 1:** Flow-chart for the SNP and sample quality control

Supplementary Figure 2: QQ plot for the univariate GWAS associations

Supplementary Figure 3: The number of associations observed for each trait at sub exome wide significance level (p<10^-4^).

Supplementary Figure 4: QQ plot for the multivariate analysis


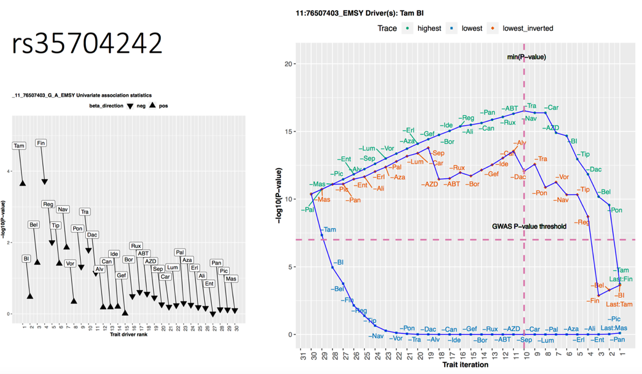


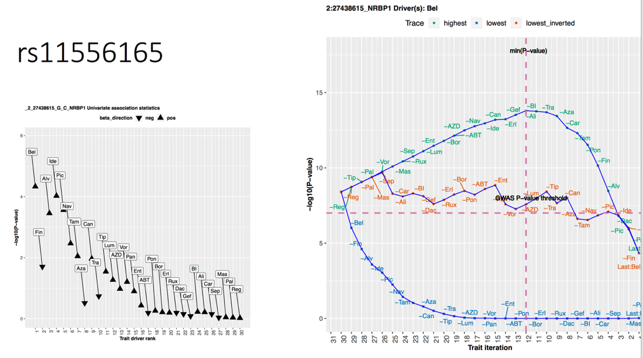


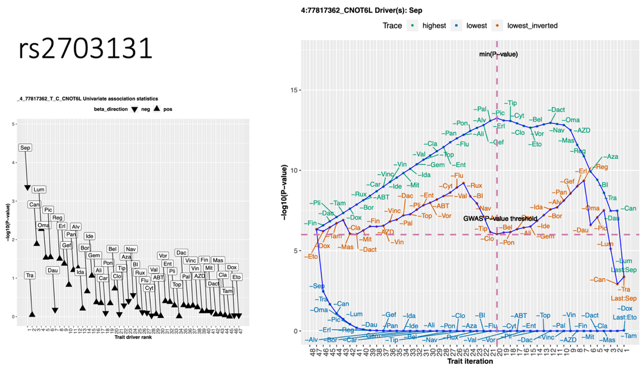


Supplementary Figure 5: QQ plot for the multivariate analysis

**Supplementary Table 1: Details of the analyzed drugs and their mechanism of action**

| **Trait** | **Healthy (N=17)** | **Diagnosis (N=108)** | **Relapsed/refractory (n=67)** |
| --- | --- | --- | --- |
| Culture medium (MCM/CM) | 0/17 | 82/26 | 46/21 |
| Age years (median, range) | 65 (19-78) | 64(21-81) | 60(19-76) |
| Sex (M/F/NA) | 4/6/7 | 45/55/8 | 20/23/24 |

NA: Not available,

**Supplementary Table 2: Details of the analyzed drugs and their mechanism of action**

| **SN** | **Drug** | **Mechanism/Targets** | **Class explained** | **Approval status** | **Solvent** | **High conc**  **(nM)** |  |
| --- | --- | --- | --- | --- | --- | --- | --- |
| 1 | Tamoxifen | Estrogen receptor antagonist | Hormone therapy | Approved | DMSO | 10000 | - |
| 2 | Navitoclax | Bcl-2/Bcl-xL inhibitor | G. Apoptotic modulator | Investigational (Ph 2) | DMSO | 10000 | NCT05222984 |
| 3 | Belinostat | HDAC inhibitor | Differentiating/ epigenetic modifier | Approved (US) | DMSO | 10000 | NCT00878722,NCT00357032 |
| 4 | Dactolisib | mTOR/(PI3K) inhibitor | Kinase inhibitor | Investigational (Ph 2) | DMSO | 1000 | NCT01756118 |
| 5 | Cladribine | Antimetabolite; Purine analog | Conv. Chemo | Approved | DMSO | 1000 | NCT00126321 |
| 6 | Idarubicin | Topoisomerase II inhibitor | Conv. Chemo | Approved | DMSO | 1000 | NCT01518556 |
| 7 | Panobinostat | HDAC inhibitor | Differentiating/ epigenetic modifier | Approved | DMSO | 1000 | NCT04326764, NCT00840346 |
| 8 | Clofarabine | Antimetabolite; Purine analog | Conv. Chemo | Approved | DMSO |  | NCT01422603,NCT00529360 |
| 9 | Fludarabine | Antimetabolite; Purine analog | Conv. Chemo | Approved | DMSO | 10000 | NCT05712278,NCT00925873 |
| 10 | Vorinostat | HDAC inhibitor | Differentiating/ epigenetic modifier | Approved | DMSO | 10000 | NCT00305773,NCT01039363 |
| 11 | Bortezomib | Proteasome inhibitor (26S subunit) | Protease/proteasome inhibitor | Approved | DMSO |  | NCT00382954,NCT01736943 |
| 12 | Tipifarnib | Farnesyltransferase inhibitor | Differentiating/ epigenetic modifier | Investigational (Ph 3) | DMSO | 10000 | NCT00048503 |
| 13 | Ponatinib | Broad TK inhibitor | Kinase inhibitor | Approved | DMSO | 1000 | NCT02428543 |
| 14 | Cytarabine | Antimetabolite, interferes with DNA synthesis | Conv. Chemo | Approved | DMSO |  | NCT00495287,NCT00464217 |
| 15 | Carfilzomib | Proteasome inhibitor (20S subunit) | L. Protease/proteasome inhibitor | Approved | DMSO | 1000 | NCT01137747,NCT02551718 |
| 16 | Etoposide | Topoisomerase II inhibitor | Conv. Chemo | Approved | DMSO | 10000 | NCT03839446,NCT00315705 |
| 17 | Idelalisib | PI3K inhibitor, p110?-selective | Kinase inhibitor | Approved | DMSO | 10000 | NCT01620216 |
| 18 | Palbociclib | CDK4/6 inhibitor | Kinase inhibitor | Approved | AQ | 10000 | NCT03844997,NCT05627232 |
| 19 | Mitoxantrone | Topoisomerase II inhibitor | Conv. Chemo | Approved | DMSO | 1000 | NCT06345365 |
| 20 | Ruxolitinib | JAK1&2 inhibitor | Kinase inhibitor | Approved | DMSO | 10000 | NCT03286530,NCT06128070 |
| 21 | Topotecan | Topoisomerase I inhibitor. Camptothecin analog | Conv. Chemo | Approved | DMSO | 10000 | NCT00488709 |
| 22 | Canertinib | pan-HER inhibitor | Kinase inhibitor | Investigational (Ph 3) | DMSO | 10000 | - |
| 23 | Valrubicin | Topoisomerase II inhibitor | Conv. Chemo | Approved | DMSO | 5000 | - |
| 24 | Vincristine | Mitotic inhibitor. Vinca alkaloid microtubule depolymerizer | Conv. Chemo | Approved | DMSO | 1000 | NCT00136084 |
| 25 | Dactinomycin | RNA and DNA synthesis inhibitor | Conv. Chemo | Approved | DMSO | 1000 | - |
| 26 | Daunorubicin | Topoisomerase II inhibitor | Conv. Chemo | Approved | DMSO | 1000 | NCT03298984 |
| 27 | Doxorubicin | Topoisomerase II inhibitor | Conv. Chemo | Approved | DMSO | 1000 | NCT01736943 |
| 28 | Entinostat | HDAC inhibitor | Differentiating/ epigenetic modifier | Investigational (Ph 2) | DMSO | 10000 | NCT00015925 |
| 29 | Luminespib | HSP90 inhibitor | HSP inhibitor | Investigational (Ph 2) | DMSO | 1000 | - |
| 30 | Fingolimod | S1PR antagonist | Other | Approved | DMSO | 10000 | - |
| 31 | Sepantronium bromide | Survivin inhibitor | Apoptotic modulator | Investigational (Ph 2) | DMSO | 10000 | - |
| 32 | Azacitidine | Nucleoside analog DNA methyl transferase inhibitor | Differentiating/ epigenetic modifier | Approved | DMSO | 10000 | NCT06345365 |
| 33 | BI 2536 | PLK1 inhibitor | Kinase inhibitor | Investigational (Ph 2) | DMSO | 1000 | NCT00701766 |
| 34 | Masitinib | KIT inhibitor | Kinase inhibitor | Investigational (Ph 3) | DMSO | 10000 | - |
| 35 | Omacetaxine | Protein synthesis inhib (80 S ribosome) | Conv. Chemo | Approved | DMSO | 10000 | NCT01272245 |
| 36 | Pictilisib | PI3K inhibitor, pan-class I | Kinase inhibitor | Investigational (Ph 2) | DMSO | 10000 | - |
| 37 | Alisertib | Aurora A inhibitor | Kinase inhibitor | Investigational (Ph 3) | DMSO | 10000 | NCT01779843 |
| 38 | Erlotinib | EGFR inhibitor | Kinase inhibitor | Approved | DMSO | 10000 | NCT01174043 |
| 39 | Plicamycin | RNA synthesis inhibitor | Conv. Chemo | Approved | DMSO |  | - |
| 40 | Vinorelbine | Mitotic inhibitor. Vinca alkaloid microtubule depolymerizer | Conv. Chemo | Approved | DMSO |  | - |
| 41 | Gefitinib | EGFR inhibitor | Kinase inhibitor | Approved | DMSO | 10000 | NCT00130702 |
| 42 | Regorafenib | B-Raf, c-Kit, VEGFR2 inhibitor | Kinase inhibitor | Approved | DMSO | 10000 | NCT06454409 |
| 43 | Trametinib | MEK1/2 inhibitor | Kinase inhibitor | Approved | DMSO | 250 | NCT04487106 |
| 44 | ABT-751 | Mitotic inhibitor. Colchicine site binding microtubule depolymerizer. | Conv. Chemo | Investigational (Ph 2) | DMSO | 10000 | - |
| 45 | AZD1775 | Wee1 inhibitor | Kinase inhibitor | Investigational (Ph 2) | DMSO | 10000 | NCT02666950 |
| 46 | Gemcitabine | Antimetabolite; Nucleoside analog | Conv. Chemo | Approved | DMSO | 1000 | NCT00268242 |
| 47 | Alvocidib | CDK inhibitor | Kinase inhibitor | Investigational (Ph 2) | DMSO | 10000 | NCT03441555,NCT03298984 |

Supplementary Table 3: SNPs showing association in the single trait analysis with *ex-vivo* drug response at exome-wide significance levels.

| **CHR** | **POS** | **SNP** | **Position** | **Shift/**  **Polyphen 2** | **Gene** | **A1/A2** | **N** | **MAF** | **Effect**  **size**  **(SE)** | **P** | **Phenotype** | **Mechanism** | **FDA**  **Approval**  **status** |
| --- | --- | --- | --- | --- | --- | --- | --- | --- | --- | --- | --- | --- | --- |
| 14 | 61446147 | rs115400838 | Missence  R (Ser) > P (Cys) | *Deleterious*  */NA* | *TRMT5* | A/T | 99 | 0.002 | 9.07  (1.54) | 8.72x10^-8^ | Idelalisib | PI3K inhibitor | Approved |
| 6 | 732629809 | rs701564 | R (Arg) > L (Leu) | *NA/NA* | *HLA-DQB1* | T/C | 99 | 0.090 | -7.42  (1.3) | 1.72x10^-7^ | Luminespib | HSP inhibitor | Investigational  (Ph 2) |
| 2 | 11414237 | rs2306234 | Missense  T (Thr) > I (Ille) | *Tolerated/*  *NA* | *BLK* | T/C | 99 | 0.160 | -7.68  (1.39) | 3.27x10^-7^ | Sepantronium bromide | Survivin inhibitor | Investigational  (Ph2)  (Ph 2) |
| 8 | 82670771 | rs35094336 | Intron variant | *Deleterious/*  *Probably*  *damaging* | *CHMPC4* | A/G | 99 | 0.05 | -4.03  (0.81) | 8.95x10^-7^ | Fludarabine | Purine analog | Approved |

*Chromosomal positions of SNPs are based on National Center for Biotechnology Information genome build 38. The effect size was calculated with respect to the minor alleles. The effect predictions for each SNPs were obtained from* Shift (https://sift.bii.a-star.edu.sg/) and Polyphen 2 (http://genetics.bwh.harvard.edu/pph2/) websites**.** *The association results presented were obtained from genotyped data in 175 patients .SE: standard error*

**Supplementary Table 4:** SNPs showing sub-exome wide significance (p<10^-4^) level association with *ex-vivo* response of drugs in the single variant analysis.

| **SN** | **chr** | **POS** | **SNP** | **Gene** | **A1/A2** | **MAF** | **N** | **BETA** | **SE** | **P** | **Drug** |
| --- | --- | --- | --- | --- | --- | --- | --- | --- | --- | --- | --- |
| 1 | 16 | 89940166 | rs139741086 | TCF25 | G/T | 0.03 | 174 | 10.24 | 2.0173 | 1.01x10-06 | Ruxolitinib |
| 2 | 5 | 7743787 | rs62342477 | ADCY2 | T/C | 0.42 | 175 | 3.89 | 0.77363 | 1.25 x10-06 | Sepantronium |
| 3 | 6 | 36343721 | rs2234075 | ETV7 | G/A | 0.01 | 174 | -9.01 | 1.81208 | 1.64 x10-06 | Belinostat |
| 4 | 5 | 150646888 | rs61740602 | GM2A | T/C | 0.10 | 175 | 6.62 | 1.33453 | 1.71 x10-06 | Alvocidib |
| 5 | 17 | 3486702 | rs224534 | TRPV1 | G/A | 0.47 | 175 | -2.11 | 0.430525 | 2.20 x10-06 | Belinostat |
| 6 | 6 | 29069264 | rs2394516 | OR2J1 | C/T | 0.38 | 175 | -2.07 | 0.422287 | 2.33 x10-06 | Cladribine |
| 7 | 6 | 29069299 | rs2394517 | OR2J1 | T/C | 0.38 | 175 | -2.07 | 0.422287 | 2.33 x10-06 | Cladribine |
| 8 | 6 | 29069016 | rs3131088 | OR2J1 | A/G | 0.38 | 175 | -2.07 | 0.422287 | 2.33 x10-06 | Cladribine |
| 9 | 3 | 63981635 | rs3733125 | ATXN7 | C/T | 0.13 | 175 | 3.49 | 0.718057 | 2.60 x10-06 | Cytarabine |
| 10 | 19 | 1924189 | rs75734024 | SCAMP4 | C/T | 0.01 | 174 | -9.92 | 2.04163 | 2.70 x10-06 | Panobinostat |
| 11 | 11 | 124267148 | rs77374761 | OR8B3 | C/T | 0.28 | 175 | -1.59 | 0.330076 | 3.18 x10-06 | ABT 751 |
| 12 | 1 | 9780836 | rs201274224 | PIK3CD | T/G | 0.03 | 174 | -5.01 | 1.0416 | 3.30 x10-06 | Daunorubicin |
| 13 | 5 | 140188709 | rs147674000 | PCDHA1 | G/T | 0.01 | 173 | 15.09 | 3.13424 | 3.31 x10-06 | Vincristine |
| 14 | 16 | 89764357 | rs150653847 | SPATA2L | A/G | 0.03 | 173 | 9.64 | 2.02419 | 4.14 x1006 | Ruxolitinib |
| 15 | 16 | 89790059 | rs4516230 | ZNF276 | G/A | 0.03 | 172 | 9.63 | 2.03091 | 4.52 x10-06 | Ruxolitinib |
| 16 | 21 | 32639157 | rs142183230 | TIAM1 | C/T | 0.01 | 174 | 6.23 | 1.31629 | 4.64 x10-06 | Azacitidine |
| 17 | 19 | 43702335 | rs140068216 | PSG4 | C/G | 0.02 | 173 | 3.64 | 0.770511 | 4.90 x10-06 | Tamoxifen |
| 18 | 6 | 29069264 | rs2394516 | OR2J1 | C/T | 0.38 | 175 | -2.72 | 0.577966 | 5.08 x10-06 | Cladribine |
| 19 | 6 | 29069299 | rs2394517 | OR2J1 | T/C | 0.38 | 175 | -2.72 | 0.577966 | 5.08 x10-06 | Cladribine |
| 20 | 6 | 29069016 | rs3131088 | OR2J1 | A/G | 0.38 | 175 | -2.72 | 0.577966 | 5.08 x10-06 | Cladribine |
| 21 | 19 | 52658231 | rs61744685 | ZNF836 | C/T | 0.03 | 175 | 4.20 | 0.903054 | 6.71 x10-06 | Azacitidine |
| 22 | 3 | 50144951 | rs1061474 | RBM5 | C/T | 0.34 | 175 | -3.91 | 0.843793 | 7.02 x10-06 | Sepantronium |
| 23 | 2 | 42515437 | rs28651764 | EML4 | A/G | 0.31 | 175 | -2.09 | 0.451982 | 7.30 x10-06 | Cladribine |
| 24 | 4 | 85414643 | rs147737825 | NKX6-1 | C/T | 0.01 | 175 | 12.78 | 2.76872 | 7.35 x10-06 | Trametinib |
| 25 | 9 | 85863091 | rs10114696 | FRMD3 | A/G | 0.35 | 175 | 2.79 | 0.60249 | 7.43 x10-06 | Entinostat |
| 26 | 17 | 45219299 | rs113472284 | CDC27 | G/A | 0.13 | 175 | 3.36 | 0.731188 | 8.468 x10-06 | Mitoxantrone |
| 27 | 3 | 128627887 | rs4494951 | ACAD9 | G/A | 0.05 | 175 | 5.04 | 1.1008 | 8.86 x10-06 | Mitoxantrone |
| 28 | 2 | 26537317 | rs150700942 | GPR113 | T/C | 0.03 | 175 | 6.38 | 1.39257 | 9.02 x10-06 | Dactolisib |
| 29 | 1 | 94343233 | rs3789457 | DNTTIP2 | C/T | 0.39 | 175 | 1.68 | 0.366324 | 9.10 x10-06 | ABT 751 |
| 30 | 10 | 3172121 | rs1052333 | PFKP | C/T | 0.39 | 175 | -3.13 | 0.68556 | 9.16 x10-06 | Canertinib |
| 31 | 18 | 77246527 | rs200207441 | NFATC1 | C/T | 0.04 | 174 | -5.97 | 1.30571 | 9.37 x10-06 | Regorafenib |
| 32 | 10 | 72015409 | rs10999212 | NPFFR1 | C/A | 0.38 | 173 | 1.44 | 0.315264 | 9.49 x10-06 | Canertinib |
| 33 | 18 | 50976898 | rs35891220 | DCC | G/A | 0.03 | 175 | 9.49 | 2.07884 | 9.77 x10-06 | Ruxolitinib |
| 34 | 17 | 3486702 | rs224534 | TRPV1 | G/A | 0.47 | 175 | -1.97 | 0.431822 | 1.02 x10-05 | Belinostat |
| 35 | 17 | 61607708 | rs7221517 | KCNH6 | T/C | 0.32 | 175 | -1.70 | 0.374366 | 1.04 x10-05 | ABT 751 |
| 36 | 5 | 150646888 | rs61740602 | GM2A | T/C | 0.10 | 175 | 4.63 | 1.01862 | 1.05 x10-05 | Alvocidib |
| 37 | 2 | 182981968 | rs1882212 | PPP1R1C | A/G | 0.17 | 175 | -2.94 | 0.650471 | 1.17 x10-05 | Erlotinib |
| 38 | 16 | 4519439 | rs11557236 | NMRAL1 | G/A | 0.10 | 175 | -5.21 | 1.1526 | 1.17 x10-05 | Topotecan |
| 39 | 5 | 140255271 | rs17844351 | PCDHA1 | G/C | 0.01 | 175 | 12.50 | 2.76872 | 1.18 x10-05 | Vincristine |
| 40 | 12 | 88481632 | rs117122459 | CEP290 | T/C | 0.05 | 175 | -4.60 | 1.01985 | 1.22 x10-05 | Vorinostat |
| 41 | 10 | 102676996 | rs41291454 | FAM178A | C/T | 0.02 | 175 | 10.38 | 2.30431 | 1.24 x10-05 | Ruxolitinib |
| 42 | 2 | 179203797 | rs1434087 | OSBPL6 | A/G | 0.08 | 175 | -4.73 | 1.04991 | 1.25 x10-05 | Alisertib |
| 43 | 14 | 39777676 | rs1950952 | MIA2 | C/G | 0.33 | 175 | -1.23 | 0.273735 | 1.26 x10-05 | Gefitinib |
| 44 | 1 | 10473200 | rs41289075 | PGD | G/A | 0.04 | 175 | 2.83 | 0.631945 | 1.39 x10-05 | Gefitinib |
| 45 | 3 | 56330063 | rs75898504 | ERC2 | T/A | 0.02 | 175 | 11.50 | 2.56859 | 1.40 x10-05 | Trametinib |
| 46 | 10 | 3172121 | rs1052333 | PFKP | C/T | 0.39 | 175 | -2.88 | 0.644659 | 1.412 x10-05 | Canertinib |
| 47 | 19 | 56320663 | rs12461110 | NLRP11 | G/A | 0.34 | 175 | 2.04 | 0.457903 | 1.44 x10-05 | Cladribine |
| 48 | 11 | 10655609 | rs2162044 | MRVI1 | T/C | 0.03 | 175 | 5.11 | 1.14473 | 1.45 x10-05 | ABT 751 |
| 49 | 1 | 158369064 | rs41488350 | OR10T2 | A/G | 0.09 | 175 | 4.24 | 0.951004 | 1.51 x10-05 | AZD1775 |
| 50 | 1 | 158368964 | rs61818749 | OR10T2 | C/T | 0.09 | 175 | 4.24 | 0.951004 | 1.51 x10-05 | AZD1775 |
| 51 | 1 | 158368974 | rs61818750 | OR10T2 | A/G | 0.09 | 175 | 4.24 | 0.951004 | 1.51 x10-05 | AZD1775 |
| 52 | 3 | 50114515 | rs7061 | RBM6 | T/C | 0.35 | 175 | -3.80 | 0.856393 | 1.60 x10-05 | Sepantronium |
| 53 | 19 | 57058998 | rs34136271 | ZFP28 | C/G | 0.13 | 175 | 4.31 | 0.972371 | 1.64 x10-05 | Trametinib |
| 54 | 1 | 9780836 | rs201274224 | PIK3CD | T/G | 0.03 | 174 | -8.47 | 1.90938 | 1.64 x10-05 | Daunorubicin |
| 55 | 6 | 36343721 | rs2234075 | ETV7 | G/A | 0.01 | 174 | -8.10 | 1.82689 | 1.68 x10-05 | Belinostat |
| 56 | 11 | 5629607 | rs10769121 | TRIM6 | G/A | 0.22 | 175 | 2.08 | 0.469568 | 1.71 x10-05 | Bortezomib |
| 57 | 1 | 159897638 | rs77570237 | IGSF9 | T/C | 0.02 | 175 | 10.43 | 2.35773 | 1.74 x10-05 | Ruxolitinib |
| 58 | 6 | 160670282 | rs316019 | SLC22A2 | C/A | 0.10 | 175 | -3.19 | 0.723093 | 1.77 x10-05 | Masitinib |
| 59 | 13 | 111335406 | rs146773721 | CARS2 | C/T | 0.03 | 175 | -5.96 | 1.34991 | 1.83 x10-05 | Daunorubicin |
| 60 | 19 | 51132746 | rs61746970 | SYT3 | G/A | 0.05 | 175 | 6.82 | 1.55013 | 1.89 x10-05 | Omacetaxine |
| 61 | 6 | 31323321 | rs1050723 | HLA-B | G/A | 0.06 | 175 | -4.49 | 1.02017 | 1.91 x10-05 | Dactolisib |
| 62 | 1 | 23418803 | rs35645814 | LUZP1 | G/C | 0.01 | 175 | 10.60 | 2.41219 | 1.94 x10-05 | Clofarabine |
| 63 | 1 | 12082334 | rs11553925 | MIIP | A/T | 0.28 | 174 | 1.05 | 0.239787 | 1.94 x10-05 | Fingolimod |
| 64 | 7 | 134618496 | rs61757650 | CALD1 | G/A | 0.03 | 175 | 7.44 | 1.69289 | 1.95 x10-05 | Clofarabine |
| 65 | 4 | 44176960 | rs13143747 | KCTD8 | T/C | 0.08 | 175 | -3.52 | 0.801503 | 1.97 x10-05 | Daunorubicin |
| 66 | 19 | 57036678 | rs3752177 | ZNF471 | G/A | 0.14 | 175 | 4.15 | 0.944841 | 1.97 x10-05 | Trametinib |
| 67 | 7 | 101892229 | rs149171097 | CUX1 | C/A | 0.03 | 172 | 3.51 | 0.801913 | 2.10 x10-05 | Azacitidine |
| 68 | 3 | 50153356 | rs1138536 | RBM5 | C/T | 0.35 | 175 | -3.69 | 0.843071 | 2.13 x10-05 | Sepantronium |
| 69 | 2 | 20494189 | rs34032508 | PUM2 | T/C | 0.02 | 175 | 8.49 | 1.94851 | 2.13 x10-05 | Ruxolitinib |
| 70 | 17 | 78176044 | rs117918077 | CARD14 | C/T | 0.02 | 175 | 8.93 | 2.05308 | 2.34 x10-05 | BI 2536 |
| 71 | 21 | 46927489 | rs12483761 | COL18A1 | A/G | 0.19 | 175 | 3.49 | 0.804165 | 2.37 x10-05 | Palbociclib |
| 72 | 7 | 151962265 | rs201834857 | MLL3 | C/T | 0.11 | 175 | -4.49 | 1.0323 | 2.37 x10-05 | Idarubicin |
| 73 | 13 | 27250823 | rs3764108 | WASF3 | T/C | 0.28 | 175 | 3.27 | 0.751919 | 2.38 x10-05 | Tipifarnib |
| 74 | 17 | 56386732 | rs61741210 | BZRAP1 | C/T | 0.03 | 175 | -4.27 | 0.981987 | 2.42 x10-05 | Palbociclib |
| 75 | 17 | 56405046 | rs61743284 | BZRAP1 | C/T | 0.03 | 175 | -4.27 | 0.981987 | 2.42 x10-05 | Palbociclib |
| 76 | 8 | 25174616 | rs142262406 | DOCK5 | C/G | 0.01 | 175 | 13.09 | 3.01538 | 2.44 x10-05 | Ruxolitinib |
| 77 | 1 | 53676448 | rs1799821 | CPT2 | A/G | 0.40 | 175 | 1.45 | 0.333593 | 2.44 x10-05 | Canertinib |
| 78 | 1 | 156255456 | rs6684514 | TMEM79 | G/A | 0.29 | 174 | 3.42 | 0.788271 | 2.44 x10-05 | Luminespib |
| 79 | 3 | 40251392 | rs1799418 | MYRIP | C/T | 0.43 | 175 | 2.92 | 0.67321 | 2.48 x10-05 | Omacetaxine |
| 80 | 3 | 128758556 | rs75140996 | CCDC48 | G/C | 0.05 | 173 | 4.80 | 1.10791 | 2.51 x10-05 | Mitoxantrone |
| 81 | 2 | 85826721 | rs11891495 | TMEM150A | G/A | 0.23 | 175 | 2.79 | 0.645287 | 2.55 x10-05 | Dactinomycin |
| 82 | 12 | 104100617 | rs697212 | STAB2 | T/C | 0.36 | 175 | 2.17 | 0.501237 | 2.59 x10-05 | Gemcitabine |
| 83 | 14 | 24035079 | rs140983547 | AP1G2 | C/T | 0.03 | 175 | 3.84 | 0.887515 | 2.60 x10-05 | Alvocidib |
| 84 | 20 | 62642781 | rs111366685 | ZNF512B | G/A | 0.03 | 175 | 8.17 | 1.88939 | 2.630 x10-05 | Trametinib |
| 85 | 2 | 182981968 | rs1882212 | PPP1R1C | A/G | 0.17 | 175 | -1.66 | 0.384976 | 2.64 x10-05 | Erlotinib |
| 86 | 5 | 150908812 | rs7718054 | FAT2 | C/T | 0.05 | 175 | 2.75 | 0.636429 | 2.64 x10-05 | Azacitidine |
| 87 | 9 | 73233933 | rs139079542 | TRPM3 | C/T | 0.01 | 175 | 12.49 | 2.89132 | 2.65 x10-05 | Trametinib |
| 88 | 7 | 151962265 | rs201834857 | MLL3 | C/T | 0.11 | 175 | -1.49 | 0.346761 | 2.66 x10-05 | Idarubicin |
| 89 | 2 | 179644035 | rs1552280 | TTN | A/G | 0.05 | 175 | -5.54 | 1.28301 | 2.67 x10-05 | Alisertib |
| 90 | 12 | 120954490 | rs3742049 | COQ5 | C/T | 0.15 | 175 | 3.68 | 0.852935 | 2. x10-05 | Idarubicin |
| 91 | 1 | 1960674 | rs28408173 | GABRD | C/T | 0.18 | 174 | 3.51 | 0.814041 | 2.71 x10-05 | Ruxolitinib |
| 92 | 19 | 55144147 | rs74906426 | LILRB1 | C/T | 0.04 | 172 | 6.18 | 1.43689 | 2.86 x10-05 | Mitoxantrone |
| 93 | 17 | 34328461 | rs854625 | CCL14 | G/A | 0.09 | 175 | -2.83 | 0.658233 | 2.91 x10-05 | Bortezomib |
| 94 | 4 | 110772711 | rs17040904 | LRIT3 | C/T | 0.09 | 175 | 4.11 | 0.957853 | 3.03 x10-05 | Dactinomycin |
| 95 | 12 | 11286249 | rs77777159 | TAS2R30 | T/C | 0.12 | 175 | 3.29 | 0.76965 | 3.06 x10-05 | Vorinostat |
| 96 | 1 | 55352613 | rs79857573 | DHCR24 | G/A | 0.02 | 175 | 7.82 | 1.82689 | 3.08 x10-05 | Dactolisib |
| 97 | 21 | 32493031 | rs762194 | TIAM1 | G/A | 0.44 | 175 | -3.03 | 0.707884 | 3.14 x10-05 | Topotecan |
| 98 | 18 | 55335366 | rs28424873 | ATP8B1 | G/T | 0.20 | 175 | -3.04 | 0.71231 | 3.16 x10-05 | Belinostat |
| 99 | 3 | 57431945 | rs62622492 | DNAH12 | T/C | 0.06 | 175 | -5.93 | 0.70 | 3.19 x10-05 | Doxorubicin |
| 100 | 22 | 19470249 | rs13447203 | CDC45 | G/A | 0.01 | 175 | 5.42 | 1.26736 | 3.21 x10-05 | Canertinib |
| 101 | 1 | 41300682 | rs55925184 | KCNQ4 | C/T | 0.05 | 175 | -4.33 | 1.01397 | 3.23 x10-05 | Panobinostat |
| 102 | 5 | 5190182 | rs34105281 | ADAMTS16 | C/T | 0.01 | 175 | 11.03 | 2.58438 | 3.26 x10-05 | Trametinib |
| 103 | 6 | 157743741 | rs187769654 | TMEM242 | T/C | 0.02 | 175 | 9.24 | 2.1639 | 3.27 x10-05 | Ruxolitinib |
| 104 | 3 | 57139972 | rs1545981 | IL17RD | G/A | 0.08 | 175 | 5.26 | 1.23406 | 3.30 x10-05 | Luminespib |
| 105 | 10 | 75533448 | rs3740289 | FUT11 | A/G | 0.03 | 175 | 7.36 | 1.72594 | 3.30 x10-05 | Alisertib |
| 106 | 20 | 33665969 | rs139800499 | TRPC4AP | C/T | 0.01 | 175 | 14.07 | 3.29915 | 3.34 x10-05 | Luminespib |
| 107 | 2 | 69709919 | rs6721259 | AAK1 | A/G | 0.15 | 175 | 3.78 | 0.886825 | 3.34 x10-05 | Vincristine |
| 108 | 20 | 62597694 | rs817329 | ZNF512B | G/T | 0.41 | 175 | -3.69 | 0.868033 | 3.41 x10-05 | Plicamycin |
| 109 | 2 | 79313990 | rs62640882 | REG1B | C/T | 0.06 | 175 | -4.17 | 0.980084 | 3.45 x10-05 | Vorinostat |
| 110 | 11 | 61089771 | rs28720291 | DDB1 | C/T | 0.01 | 175 | -9.52 | 2.23507 | 3.45 x10-05 | Etoposide |
| 111 | 11 | 10655609 | rs2162044 | MRVI1 | T/C | 0.03 | 175 | 9.22 | 2.16944 | 3.53 x10-05 | ABT 751 |
| 112 | 22 | 29446695 | rs55962108 | ZNRF3 | G/A | 0.05 | 174 | 5.01 | 1.18063 | 3.67 x10-05 | Alisertib |
| 113 | 14 | 68045935 | rs6573781 | PLEKHH1 | A/G | 0.16 | 174 | 4.48 | 1.05644 | 3.68 x10-05 | Vinorelbine |
| 114 | 17 | 34340284 | rs1003645 | CCL23 | T/C | 0.18 | 175 | -2.13 | 0.503314 | 3.68 x10-05 | Bortezomib |
| 115 | 5 | 71756670 | rs2278600 | ZNF366 | C/T | 0.16 | 174 | 1.28 | 0.302691 | 3.74 x10-05 | Fingolimod |
| 116 | 22 | 36684980 | rs11549907 | MYH9 | G/A | 0.02 | 174 | 8.79 | 2.07817 | 3.79 x10-05 | Dactinomycin |
| 117 | 11 | 71259822 | rs10792769 | KRTAP5-9 | A/G | 0.17 | 172 | -1.97 | 0.465157 | 3.84 x10-05 | Fludarabine |
| 118 | 5 | 153783753 | rs6580076 | GALNT10 | C/T | 0.15 | 174 | 3.57 | 0.845073 | 3.84 x10-05 | BI 2536 |
| 119 | 12 | 57004268 | rs34393899 | BAZ2A | G/A | 0.07 | 175 | -6.64 | 1.57047 | 3.85 x10-05 | Sepantronium |
| 120 | 1 | 10342522 | rs17034660 | KIF1B | G/A | 0.04 | 175 | 2.62 | 0.618812 | 3.88 x10-05 | Gefitinib |
| 121 | 1 | 10397567 | rs2297881 | KIF1B | A/G | 0.04 | 175 | 2.62 | 0.618812 | 3.88 x10-05 | Gefitinib |
| 122 | 1 | 205385412 | rs16855539 | LEMD1 | A/G | 0.03 | 172 | 6.26 | 1.48127 | 3.90 x10-05 | Alisertib |
| 123 | 1 | 160650938 | rs6667145 | CD48 | C/T | 0.13 | 175 | 3.04 | 0.720689 | 3.93 x10-05 | Idelalisib |
| 124 | 13 | 111335406 | rs146773721 | CARS2 | C/T | 0.03 | 175 | -5.26 | 1.24575 | 3.96 x10-05 | Daunorubicin |
| 125 | 10 | 3172121 | rs1052333 | PFKP | C/T | 0.39 | 175 | -2.06 | 0.489569 | 3.96 x10-05 | Canertinib |
| 126 | 22 | 24300540 | rs144128236 | GSTT2B | C/T | 0.01 | 173 | 8.46 | 2.00479 | 4.03 x10-05 | Erlotinib |
| 127 | 19 | 55346534 | rs112522228 | KIR3DL1 | G/C | 0.11 | 172 | -2.70 | 0.639847 | 4.04 x10-05 | Cladribine |
| 128 | 22 | 36691691 | rs875725 | MYH9 | T/C | 0.02 | 175 | 8.76 | 2.07803 | 4.08 x10-05 | Dactinomycin |
| 129 | 1 | 42896441 | rs41301052 | ZMYND12 | C/A | 0.04 | 175 | 7.23 | 1.71615 | 4.08 x10-05 | Luminespib |
| 130 | 1 | 9780836 | rs201274224 | PIK3CD | T/G | 0.03 | 174 | -8.67 | 2.05703 | 4.08 x10-05 | Daunorubicin |
| 131 | 2 | 219221846 | rs4324314 | C2orf62 | G/A | 0.19 | 175 | -2.96 | 0.702267 | 4.08 x10-05 | Dactinomycin |
| 132 | 4 | 9922170 | rs73225891 | SLC2A9 | C/G | 0.07 | 175 | -4.62 | 1.09711 | 4.15 x10-05 | Mitoxantrone |
| 133 | 1 | 9780836 | rs201274224 | PIK3CD | T/G | 0.03 | 174 | -4.39 | 1.04434 | 4.16 x10-05 | Daunorubicin |
| 134 | 14 | 24035079 | rs140983547 | AP1G2 | C/T | 0.03 | 175 | 6.27 | 1.48923 | 4.19 x10-05 | Alvocidib |
| 135 | 10 | 3172121 | rs1052333 | PFKP | C/T | 0.39 | 175 | -1.33 | 0.316233 | 4.20 x10-05 | Canertinib |
| 136 | 22 | 29446695 | rs55962108 | ZNRF3 | G/A | 0.05 | 174 | 5.28 | 1.25589 | 4.22 x10-05 | Alisertib |
| 137 | 6 | 149700491 | rs3734296 | TAB2 | G/A | 0.13 | 175 | 4.05 | 0.964672 | 4.34 x10-05 | Vincristine |
| 138 | 6 | 149730846 | rs652921 | TAB2 | G/A | 0.13 | 175 | 4.05 | 0.964672 | 4.34 x10-05 | Vincristine |
| 139 | 20 | 62595169 | rs817325 | ZNF512B | G/A | 0.40 | 174 | -3.67 | 0.875653 | 4.38 x10-05 | Plicamycin |
| 140 | 1 | 156264000 | rs10908496 | C1orf85 | G/A | 0.29 | 175 | 3.29 | 0.786347 | 4.42 x10-05 | Luminespib |
| 141 | 3 | 57431945 | rs62622492 | DNAH12 | T/C | 0.06 | 175 | -2.96 | 0.706064 | 4.43 x10-05 | Doxorubicin |
| 142 | 20 | 30915461 | rs1129012 | KIF3B | T/C | 0.17 | 175 | -1.23 | 0.293314 | 4.44 x10-05 | Fingolimod |
| 143 | 1 | 211652382 | rs143207434 | RD3 | T/A | 0.02 | 174 | 9.78 | 2.33347 | 4.46 x10-05 | Ruxolitinib |
| 144 | 3 | 128634037 | rs113633667 | ACAD9 | C/T | 0.05 | 173 | 4.66 | 1.11312 | 4.48 x10-05 | Mitoxantrone |
| 145 | 14 | 94103602 | rs4905082 | UNC79 | A/G | 0.35 | 175 | 1.91 | 0.456456 | 4.49 x10-05 | Alvocidib |
| 146 | 16 | 16139714 | rs35587 | ABCC1 | T/C | 0.31 | 175 | 1.60 | 0.382435 | 4.61 x10-05 | Carfilzomib |
| 147 | 6 | 35426175 | rs3823434 | FANCE | C/T | 0.05 | 175 | -4.87 | 1.16443 | 4.61 x10-05 | Gemcitabine |
| 148 | 2 | 27438615 | rs11556165 | C2orf28 | G/C | 0.04 | 175 | 4.42 | 1.05619 | 4.64 x10-05 | Belinostat |
| 149 | 1 | 39340862 | rs874243 | MYCBP | A/G | 0.25 | 175 | -2.17 | 0.518982 | 4.66 x10-05 | Daunorubicin |
| 150 | 15 | 85341859 | rs8182086 | ZNF592 | G/A | 0.23 | 175 | 1.89 | 0.452726 | 4.76 x10-05 | ABT 751 |
| 151 | 1 | 42630364 | rs2071499 | GUCA2A | A/G | 0.35 | 173 | -1.63 | 0.391391 | 4.76 x10-05 | Carfilzomib |
| 152 | 14 | 94103602 | rs4905082 | UNC79 | A/G | 0.35 | 175 | 3.06 | 0.734085 | 4.82 x10-05 | Alvocidib |
| 153 | 10 | 13378273 | rs62641683 | SEPHS1 | G/C | 0.03 | 175 | -6.72 | 1.61033 | 4.85 x10-05 | Gemcitabine |
| 154 | 19 | 49898404 | rs191973091 | CCDC155 | G/A | 0.02 | 174 | 3.28 | 0.78707 | 4.86 x10-05 | Fingolimod |
| 155 | 1 | 10479791 | rs41274480 | PGD | G/A | 0.04 | 175 | 2.73 | 0.656193 | 4.91 x10-05 | Gefitinib |
| 156 | 5 | 140362125 | rs79247475 | PCDHA1 | C/G | 0.02 | 175 | 8.74 | 2.09841 | 4.97 x10-05 | Luminespib |
| 157 | 17 | 3844787 | rs1800912 | ATP2A3 | C/G | 0.22 | 173 | -4.07 | 0.977207 | 4.99 x10-05 | Plicamycin |
| 158 | 2 | 96798440 | rs61735195 | ASTL | C/T | 0.03 | 175 | 4.38 | 1.0511 | 0.000050169 | Fludarabine |
| 159 | 22 | 43289473 | rs5759013 | PACSIN2 | G/A | 0.33 | 174 | -2.01 | 0.481869 | 5.02 x10-05 | Alisertib |
| 160 | 7 | 151962257 | rs62478357 | MLL3 | C/T | 0.11 | 175 | -1.45 | 0.347914 | 5.04 x10-05 | Idarubicin |
| 161 | 1 | 114438951 | rs1217401 | AP4B1 | A/G | 0.30 | 175 | 1.99 | 0.478235 | 5.05 x10-05 | Cladribine |
| 162 | 17 | 78176044 | rs117918077 | CARD14 | C/T | 0.02 | 175 | 9.55 | 2.29659 | 5.06 x10-05 | BI 2536 |
| 163 | 18 | 55335366 | rs28424873 | ATP8B1 | G/T | 0.20 | 175 | -3.28 | 0.788452 | 5.10 x10-05 | Belinostat |
| 164 | 11 | 117335686 | rs61743159 | DSCAML1 | G/A | 0.05 | 175 | 5.48 | 1.31703 | 5.13 x10-05 | Clofarabine |
| 165 | 14 | 94103602 | rs4905082 | UNC79 | A/G | 0.35 | 175 | 2.03 | 0.489657 | 5.23 x10-05 | Alvocidib |
| 166 | 22 | 24300540 | rs144128236 | GSTT2B | C/T | 0.01 | 173 | 4.97 | 1.19807 | 5.23 x10-05 | Erlotinib |
| 167 | 2 | 173352157 | rs2293648 | ITGA6 | A/T | 0.05 | 175 | -5.93 | 1.42929 | 5.25 x10-05 | BI 2536 |
| 168 | 6 | 28963248 | rs6456880 | ZNF311 | T/G | 0.38 | 175 | -1.79 | 0.432058 | 5.31 x10-05 | Cladribine |
| 169 | 16 | 1260969 | rs61910714 | CACNA1H | G/A | 0.10 | 174 | -3.72 | 0.897501 | 5.34 x10-05 | Alisertib |
| 170 | 19 | 17366278 | rs4430871 | USHBP1 | C/T | 0.40 | 173 | -2.19 | 0.530052 | 5.42 x10-05 | Alisertib |
| 171 | 11 | 124267148 | rs77374761 | OR8B3 | C/T | 0.28 | 175 | -1.75 | 0.421865 | 5.43 x10-05 | ABT 751 |
| 172 | 2 | 42515437 | rs28651764 | EML4 | A/G | 0.31 | 175 | -1.50 | 0.36251 | 5.46 x10-05 | Cladribine |
| 173 | 5 | 150646888 | rs61740602 | GM2A | T/C | 0.10 | 175 | 3.00 | 0.724778 | 5.48 x10-05 | Alvocidib |
| 174 | 10 | 14890829 | rs45567440 | HSPA14 | C/A | 0.01 | 175 | -8.49 | 2.05034 | 5.51 x10-05 | Belinostat |
| 175 | 1 | 41012847 | rs41268081 | ZNF684 | A/G | 0.07 | 175 | 3.28 | 0.792814 | 5.54 x10-05 | Masitinib |
| 176 | X | 12904662 | rs5743780 | TLR7 | G/A | 0.02 | 175 | 7.42 | 1.79282 | 5.56 x10-05 | Clofarabine |
| 177 | 21 | 32639157 | rs142183230 | TIAM1 | C/T | 0.01 | 174 | 9.25 | 1.31 | 5.62 x10-05 | Azacitidine |
| 178 | 5 | 79026187 | rs17253860 | CMYA5 | A/G | 0.01 | 175 | -7.00 | 1.69362 | 5.62 x10-05 | Carfilzomib |
| 179 | 12 | 27064232 | rs3210635 | ASUN | T/C | 0.44 | 175 | -2.52 | 0.609084 | 5.66 x10-05 | ABT 751 |
| 180 | 1 | 17660499 | rs874881 | PADI4 | C/G | 0.48 | 175 | 1.57 | 0.382264 | 5.69 x10-05 | ABT 751 |
| 181 | 16 | 84228873 | rs11865115 | ADAD2 | C/T | 0.16 | 174 | -2.47 | 0.598834 | 5.73 x10-05 | Daunorubicin |
| 182 | 1 | 215901492 | rs2820718 | USH2A | C/T | 0.21 | 175 | -1.23 | 0.297314 | 5.73 x10-05 | Gefitinib |
| 183 | 5 | 115811273 | rs17432496 | CTB-118N6.3 | C/T | 0.03 | 175 | 5.43 | 1.31591 | 5.74 x10-05 | Regorafenib |
| 184 | 1 | 156263940 | rs10908495 | C1orf85 | T/C | 0.28 | 175 | 3.24 | 0.785168 | 5.74 x10-05 | Luminespib |
| 185 | 18 | 55322502 | rs12968116 | ATP8B1 | C/T | 0.12 | 175 | 3.69 | 0.89294 | 5.79 x10-05 | AZD1775 |
| 186 | 22 | 32111553 | rs140450525 | PRR14L | G/A | 0.03 | 175 | 3.27 | 0.793453 | 5.79 x10-05 | Gefitinib |
| 187 | 1 | 89449218 | rs113402009 | CCBL2 | G/A | 0.01 | 175 | -9.68 | 2.34926 | 5.92 x10-05 | Mitoxantrone |
| 188 | 1 | 53980367 | rs142735388 | GLIS1 | G/A | 0.01 | 174 | 5.52 | 1.34 | 6.02 x10-05 | Azacitidine |
| 189 | 10 | 75533448 | rs3740289 | FUT11 | A/G | 0.03 | 175 | 6.70 | 1.62832 | 6.06 x10-05 | Alisertib |
| 190 | 4 | 139140494 | rs6838248 | SLC7A11 | G/C | 0.37 | 175 | 2.08 | 0.506795 | 6.06 x10-05 | Vorinostat |
| 191 | 21 | 46927489 | rs12483761 | COL18A1 | A/G | 0.19 | 175 | 1.79 | 0.434703 | 6.171 x10-05 | Palbociclib |
| 192 | 2 | 69709919 | rs6721259 | AAK1 | A/G | 0.15 | 175 | 4.55 | 1.10851 | 6.23 x10-05 | Vincristine |
| 193 | 14 | 94187832 | rs1887197 | PRIMA1 | T/C | 0.40 | 175 | 1.83 | 0.44549 | 6.23 x10-05 | Panobinostat |
| 194 | 19 | 53117809 | rs10406458 | ZNF83 | A/C | 0.36 | 175 | 3.08 | 0.750271 | 6.23 x10-05 | Tipifarnib |
| 195 | 12 | 57861785 | rs61734545 | GLI1 | T/C | 0.04 | 175 | -7.67 | 1.86829 | 6.26 x10-05 | Sepantronium |
| 196 | 13 | 42876290 | rs41288311 | AKAP11 | A/C | 0.17 | 175 | 2.37 | 0.576452 | 6.33 x10-05 | Belinostat |
| 197 | 22 | 24300540 | rs144128236 | GSTT2B | C/T | 0.01 | 173 | 4.28 | 1.0437 | 6.35 x10-05 | Erlotinib |
| 198 | 5 | 73090261 | rs7716253 | ARHGEF28 | C/T | 0.48 | 175 | 2.19 | 0.534259 | 6.37 x10-05 | Gemcitabine |
| 199 | 2 | 132021078 | rs200735283 | PLEKHB2 | A/G | 0.02 | 172 | 6.24 | 1.52167 | 6.39 x10-05 | Idelalisib |
| 200 | 19 | 58992029 | rs142233722 | ZNF446 | G/A | 0.01 | 172 | 12.53 | 3.05669 | 6.46 x10-05 | Ruxolitinib |
| 201 | 15 | 100211780 | rs201205600 | MEF2A | A/G | 0.01 | 175 | 13.48 | 3.28875 | 6.49 x10-05 | Luminespib |
| 202 | 15 | 91496242 | rs8041035 | AC068831.6 | C/T | 0.04 | 175 | 4.62 | 1.12803 | 6.50 x10-05 | Cladribine |
| 203 | 10 | 50667120 | rs61760167 | ERCC6 | T/G | 0.01 | 175 | 12.12 | 2.95843 | 6.51 x10-05 | BI 2536 |
| 204 | 2 | 166781158 | rs2163649 | TTC21B | G/A | 0.04 | 175 | 2.22 | 0.542945 | 6.54 x10-05 | Tamoxifen |
| 205 | 2 | 166767913 | rs80225158 | TTC21B | C/G | 0.04 | 175 | 2.22 | 0.542945 | 6.54 x10-05 | Tamoxifen |
| 206 | 2 | 136546110 | rs2278544 | LCT | G/A | 0.28 | 175 | -2.27 | 0.554641 | 6.57 x10-05 | Idelalisib |
| 207 | 8 | 18080196 | rs4986783 | NAT1 | T/G | 0.02 | 175 | 3.17 | 0.773925 | 6.57 x10-05 | Tamoxifen |
| 208 | 8 | 18080015 | rs4986990 | NAT1 | G/A | 0.02 | 175 | 3.17 | 0.773925 | 6.57 x10-05 | Tamoxifen |
| 209 | 8 | 18080001 | rs4987076 | NAT1 | G/A | 0.02 | 175 | 3.17 | 0.773925 | 6.57 x10-05 | Tamoxifen |
| 210 | 1 | 152692512 | rs41268474 | C1orf68 | G/A | 0.09 | 175 | -2.08 | 0.508526 | 6.69 x10-05 | Azacitidine |
| 211 | 19 | 37039069 | rs2912444 | ZNF529 | C/A | 0.27 | 175 | -2.06 | 0.504192 | 6.79 x10-05 | Cladribine |
| 212 | 6 | 36343721 | rs2234075 | ETV7 | G/A | 0.01 | 174 | -10.73 | 2.62717 | 6.84 x10-05 | Belinostat |
| 213 | 22 | 43203137 | rs738535 | ARFGAP3 | C/T | 0.33 | 175 | -1.98 | 0.484845 | 6.90 x10-05 | Regorafenib |
| 214 | 12 | 46589988 | rs11608815 | SLC38A1 | C/T | 0.12 | 174 | 4.89 | 1.19763 | 6.99 x10-05 | Sepantronium |
| 215 | 1 | 9780836 | rs201274224 | PIK3CD | T/G | 0.03 | 174 | -8.64 | 2.11911 | 7.00 x10-05 | Daunorubicin |
| 216 | 1 | 9780836 | rs201274224 | PIK3CD | T/G | 0.03 | 174 | -5.64 | 1.38236 | 7.00 x10-05 | Daunorubicin |
| 217 | 7 | 87138645 | rs1045642 | ABCB1 | A/G | 0.39 | 175 | 2.41 | 0.592489 | 7.04 x10-05 | Entinostat |
| 218 | 22 | 32234797 | rs61731664 | DEPDC5 | G/A | 0.03 | 175 | 3.08 | 0.755554 | 7.06 x10-05 | Gefitinib |
| 219 | 22 | 32200161 | rs79070552 | DEPDC5 | T/C | 0.03 | 175 | 3.08 | 0.755554 | 7.06 x10-05 | Gefitinib |
| 220 | 7 | 150765065 | rs2229550 | SLC4A2 | C/T | 0.17 | 174 | -3.85 | 0.944492 | 7.13 x10-05 | Omacetaxine |
| 221 | 9 | 140147765 | rs1134511 | C9orf173 | C/T | 0.03 | 173 | 6.35 | 1.55913 | 7.13 x10-05 | Entinostat |
| 222 | 12 | 27064232 | rs3210635 | ASUN | T/C | 0.44 | 175 | -1.53 | 0.37667 | 7.14 x10-05 | ABT 751 |
| 223 | 16 | 79245687 | rs199576434 | WWOX | C/T | 0.01 | 174 | -8.46 | 2.07766 | 7.18 x10-05 | Belinostat |
| 224 | 6 | 26285683 | rs2393593 | HIST1H4H | C/T | 0.37 | 175 | 2.23 | 0.545008 | 7.23 x10-05 | Dactinomycin |
| 225 | 18 | 61600384 | rs963075 | SERPINB10 | C/T | 0.28 | 175 | 2.87 | 0.706917 | 7.30 x10-05 | Idarubicin |
| 226 | 7 | 87179601 | rs1128503 | ABCB1 | G/A | 0.46 | 175 | -1.76 | 0.434256 | 7.37 x10-05 | Cladribine |
| 227 | 5 | 73931246 | rs442425 | ENC1 | C/T | 0.29 | 175 | -1.09 | 0.268149 | 7.39 x10-05 | Gefitinib |
| 228 | 1 | 42896441 | rs41301052 | ZMYND12 | C/A | 0.04 | 175 | 7.59 | 1.86918 | 7.39 x10-05 | Luminespib |
| 229 | 11 | 1018169 | rs77647814 | MUC6 | C/G | 0.05 | 175 | 4.20 | 1.05773 | 7.41 x10-05 | Vorinostat |
| 230 | 11 | 72408055 | rs56200889 | ARAP1 | G/C | 0.34 | 173 | 1.64 | 0.404591 | 7.43 x10-05 | Bortezomib |
| 231 | 7 | 142655008 | rs8176058 | KEL | G/A | 0.02 | 175 | -6.64 | 1.63483 | 7.49 x10-05 | Panobinostat |
| 232 | 5 | 35072712 | rs72478580 | PRLR | T/G | 0.02 | 175 | -6.44 | 1.59 | 7.55 x10-05 | Masitinib |
| 233 | 6 | 111628626 | rs3204953 | REV3L | C/T | 0.11 | 175 | 3.35 | 0.826582 | 7.60 x10-05 | Dactinomycin |
| 234 | 6 | 111628597 | rs3204954 | REV3L | C/G | 0.11 | 175 | 3.35 | 0.826582 | 7.60 x10-05 | Dactinomycin |
| 235 | 1 | 53980367 | rs142735388 | GLIS1 | G/A | 0.01 | 174 | 8.37 | 2.06582 | 7.71 x10-05 | Azacitidine |
| 236 | 2 | 26676349 | rs200824609 | CCDC164 | C/G | 0.01 | 175 | 12.34 | 3.04501 | 7.72 x10-05 | Ruxolitinib |
| 237 | 7 | 150695726 | rs1549758 | NOS3 | C/T | 0.28 | 173 | -2.09 | 0.516395 | 7.75 x10-05 | Dactolisib |
| 238 | 10 | 102738766 | rs118166648 | MRPL43 | T/C | 0.10 | 175 | -2.54 | 0.628189 | 7.83 x10-05 | ABT 751 |
| 239 | 16 | 111247 | rs79691782 | RHBDF1 | C/T | 0.07 | 173 | -5.34 | 1.31978 | 7.99 x10-05 | Omacetaxine |
| 240 | 5 | 64887614 | rs61748229 | TRIM23 | T/C | 0.03 | 175 | -6.81 | 1.68432 | 8.08 x10-05 | Trametinib |
| 241 | 2 | 69741801 | rs6757825 | AAK1 | C/T | 0.15 | 175 | 4.53 | 1.12155 | 8.11 x10-05 | Vinorelbine |
| 242 | 2 | 135745129 | rs16831235 | YSK4 | G/A | 0.15 | 175 | -1.47 | 0.364262 | 8.12 x10-05 | Gefitinib |
| 243 | 17 | 7484782 | rs17607 | CD68 | G/A | 0.08 | 175 | -2.17 | 0.536206 | 8.14 x10-05 | Azacitidine |
| 244 | 22 | 43289473 | rs5759013 | PACSIN2 | G/A | 0.33 | 174 | -2.14 | 0.529237 | 8.19 x10-05 | Alisertib |
| 245 | 17 | 45219299 | rs113472284 | CDC27 | G/A | 0.13 | 175 | 3.61 | 0.895523 | 8.25 x10-05 | Mitoxantrone |
| 246 | 4 | 120240238 | rs1511025 | FABP2 | T/C | 0.29 | 175 | 1.49 | 0.370131 | 8.32 x10-05 | Palbociclib |
| 247 | 17 | 76045806 | rs141190761 | TNRC6C | C/T | 0.01 | 175 | -8.33 | 2.06506 | 8.35 x10-05 | Panobinostat |
| 248 | 20 | 60768573 | rs41284984 | GTPBP5 | C/T | 0.04 | 175 | 7.64 | 1.89595 | 8.37 x10-05 | Tipifarnib |
| 249 | 7 | 101892229 | rs149171097 | CUX1 | C/A | 0.03 | 172 | 5.40 | 1.33925 | 8.43 x10-05 | Azacitidine |
| 250 | 22 | 38328597 | rs34834842 | MICALL1 | A/G | 0.03 | 175 | -5.91 | 1.46645 | 8.44 x10-05 | Regorafenib |
| 251 | 6 | 152772264 | rs214976 | SYNE1 | A/G | 0.40 | 175 | -2.41 | 0.597412 | 8.44 x10-05 | Entinostat |
| 252 | 14 | 68035879 | rs3825725 | PLEKHH1 | A/G | 0.25 | 175 | -2.57 | 0.63857 | 8.51 x10-05 | Mitoxantrone |
| 253 | 6 | 169064783 | rs118133242 | SMOC2 | A/G | 0.01 | 175 | 9.79 | 2.43404 | 8.69 x10-05 | Entinostat |
| 254 | 11 | 2924591 | rs1048046 | SLC22A18AS | G/A | 0.33 | 174 | -1.91 | 0.474677 | 8.7192 x10-05 | Panobinostat |
| 255 | 6 | 159457826 | rs144047559 | TAGAP | C/G | 0.01 | 175 | 8.29 | 2.06417 | 8.787 x10-05 | Idelalisib |
| 256 | 3 | 40286017 | rs4618168 | MYRIP | T/C | 0.43 | 175 | 2.68 | 0.666225 | 8.82 x10-05 | Omacetaxine |
| 257 | 4 | 118975146 | rs13152576 | NDST3 | C/T | 0.21 | 175 | 3.02 | 0.751172 | 8.84 x10-05 | AZD1775 |
| 258 | 20 | 60791866 | rs41284986 | HRH3 | G/A | 0.02 | 175 | -8.85 | 2.20105 | 8.84 x10-05 | Clofarabine |
| 259 | 17 | 3476990 | rs17706245 | TRPV1 | G/A | 0.04 | 175 | -4.58 | 1.14075 | 8.89 x10-05 | Masitinib |
| 260 | 17 | 3480910 | rs34378673 | TRPV1 | A/G | 0.04 | 175 | -4.58 | 1.14075 | 8.89 x10-05 | Masitinib |
| 261 | 17 | 3475490 | rs877610 | TRPV1 | C/T | 0.04 | 175 | -4.58 | 1.14075 | 8.89 x10-05 | Masitinib |
| 262 | 3 | 4856180 | rs711631 | AC018816.3 | C/T | 0.13 | 175 | 3.76 | 0.937373 | 8.96 x10-05 | Masitinib |
| 263 | 5 | 78340257 | rs248385 | DMGDH | C/G | 0.49 | 175 | -1.93 | 0.480411 | 8.96 x10-05 | Dactolisib |
| 264 | 5 | 78340286 | rs532964 | DMGDH | A/G | 0.49 | 175 | -1.93 | 0.480411 | 8.96 x10-05 | Dactolisib |
| 265 | 6 | 26033641 | rs2230654 | HIST1H2AB | G/A | 0.12 | 174 | -4.86 | 1.21046 | 8.99 x10-05 | Vinorelbine |
| 266 | 2 | 85824039 | rs1127974 | RNF181 | G/A | 0.16 | 175 | 2.92 | 0.728904 | 8.99 x10-05 | Dactinomycin |
| 267 | 3 | 4856180 | rs711631 | AC018816.3 | C/T | 0.13 | 175 | 2.41 | 0.600483 | 9.05 x10-05 | Masitinib |
| 268 | 18 | 55335366 | rs28424873 | ATP8B1 | G/T | 0.20 | 175 | -2.23 | 0.557855 | 9.06 x10-05 | Belinostat |
| 269 | 1 | 152538495 | rs141660085 | LCE3E | G/A | 0.02 | 174 | 4.34 | 1.08302 | 9.09 x10-05 | Erlotinib |
| 270 | 1 | 53600078 | rs3737989 | SLC1A7 | A/G | 0.15 | 175 | 3.30 | 0.824444 | 9.11 x10-05 | Clofarabine |
| 271 | 1 | 22154845 | rs2228347 | HSPG2 | A/G | 0.35 | 175 | -3.77 | 0.939706 | 9.21 x10-05 | Navitoclax |
| 272 | 22 | 38470909 | rs200682835 | PICK1 | A/T | 0.01 | 175 | 10.16 | 2.53611 | 9.22 x10-05 | Dactinomycin |
| 273 | 1 | 228871693 | rs3738073 | RHOU | C/T | 0.19 | 173 | -1.83 | 0.457717 | 9.22 x10-05 | Doxorubicin |
| 274 | 11 | 60102507 | rs2304933 | MS4A6E | T/G | 0.49 | 175 | 2.27 | 0.566338 | 9.26 x10-05 | BI 2536 |
| 275 | 11 | 60102396 | rs2304934 | MS4A6E | G/A | 0.49 | 175 | 2.27 | 0.566338 | 9.26 x10-05 | BI 2536 |
| 276 | 11 | 60102384 | rs2304935 | MS4A6E | G/A | 0.49 | 175 | 2.27 | 0.566338 | 9.26 x10-05 | BI 2536 |
| 277 | 7 | 151962257 | rs62478357 | MLL3 | C/T | 0.11 | 175 | -4.17 | 1.04007 | 9.33 x10-05 | Idarubicin |
| 278 | 2 | 27438615 | rs11556165 | C2orf28 | G/C | 0.04 | 175 | 4.75 | 1.18635 | 9.39 x10-05 | Belinostat |
| 279 | 12 | 117160976 | rs10507274 | C12orf49 | T/C | 0.05 | 175 | -5.20 | 1.29927 | 9.40 x10-05 | Entinostat |
| 280 | 1 | 41486245 | rs1138293 | SLFNL1 | C/T | 0.20 | 175 | -4.53 | 1.13276 | 9.45 x10-05 | Navitoclax |
| 281 | 19 | 18375608 | rs8102923 | KIAA1683 | G/A | 0.20 | 175 | -2.65 | 0.663864 | 9.50 x10-05 | Gemcitabine |
| 282 | 22 | 40075341 | rs59504308 | CACNA1I | C/T | 0.03 | 173 | -7.79 | 1.94851 | 9.56 x10-05 | Idarubicin |
| 283 | 4 | 57889677 | rs1056364 | POLR2B | T/C | 0.45 | 175 | 0.84 | 0.211006 | 9.61 x10-05 | Fingolimod |
| 284 | 2 | 103335646 | rs115622745 | MFSD9 | A/G | 0.02 | 174 | 6.27 | 1.56948 | 9.78 x10-05 | Belinostat |
| 285 | 17 | 34341398 | rs61737011 | CCL23 | A/G | 0.05 | 175 | -3.62 | 0.906347 | 9.79 x10-05 | Bortezomib |
| 286 | 1 | 183072701 | rs2296289 | LAMC1 | C/T | 0.13 | 175 | 4.05 | 1.01454 | 9.81 x10-05 | Luminespib |
| 287 | 9 | 137593099 | rs138579182 | COL5A1 | G/A | 0.05 | 175 | 6.07 | 1.52006 | 9.81 x10-05 | Pictilisib |
| 288 | 1 | 186022964 | rs144191448 | HMCN1 | A/G | 0.02 | 175 | 3.11 | 0.779314 | 9.82 x10-05 | Fingolimod |
| 289 | 1 | 186031713 | rs148467349 | HMCN1 | G/A | 0.02 | 175 | 3.11 | 0.779314 | 9.82 x10-05 | Fingolimod |
| 290 | 18 | 47796017 | rs72923678 | MBD1 | G/A | 0.01 | 175 | -8.17 | 2.048 | 9.84 x10-05 | Panobinostat |
| 291 | 16 | 79245687 | rs199576434 | WWOX | C/T | 0.01 | 174 | -8.35 | 2.09269 | 9.85 x10-05 | Belinostat |
| 292 | 11 | 1263687 | rs55933911 | MUC5B | C/T | 0.11 | 175 | 2.25 | 0.563395 | 9.87 x10-05 | Palbociclib |
| 293 | 14 | 24035079 | rs140983547 | AP1G2 | C/T | 0.03 | 175 | 3.11 | 0.780317 | 9.87 x10-05 | Alvocidib |
| 294 | 3 | 36931397 | rs74424604 | TRANK1 | C/T | 0.03 | 175 | 6.05 | 1.52 | 9.91 x10-05 | Valrubicin |
| 295 | 4 | 183664522 | rs7654255 | ODZ3 | T/C | 0.33 | 175 | 2.10 | 0.526866 | 9.99 x10-05 | Alvocidib |

**Supplementary Table 5:** SNPs showing association with *ex-vivo* response of all 47 drugs as a group at the genome-wide significance levels in the multivariate analysis.

| **CHR** | **BP** | **Variant (Gene)** | **Allele** | **MAF** | **Pval** | **Central traits** | **Drug group** |
| --- | --- | --- | --- | --- | --- | --- | --- |
| 11 | 76507403 | rs11236938-G/A (*TSKU*) | S (Ser) > N (Asn) | 0.03 | 2.51x10-9 | Tamoxifen, BI.2536, Belinostat | All 47 drugs |
| 2 | 27438615 | rs11556165-G/C(*ATRAID*) | D (Asp) > H (His) | 0.04 | 4.26x10-8 | Belinostat, Fingolimod, Alvocidib | All 47 drugs |

**Supplementary Table 6:** SNPs showing association with random *ex-vivo* response of non-chemo drugs as a group at the genome-wide significance levels in the multivariate analysis.

| **Variant** | **Allele** | **Freq** | **Driver traits** | **Kinase only (17)** |
| --- | --- | --- | --- | --- |
| rs8068970-C/T (KRT40) | Q (Gln) > Q (Gln) | 0.23 | Alvocidib, Dactolisib, Entinostat | 8.23 |
|  |  |  |  |  |
| rs34559356-T/C(KCNG4) | A (Ala) > A (Ala) | 0.03 | Bortezomib, AZD1775, Alisertib | 8.20 |
| rs62344598-G/A (LRBA) | L (Leu) > M (Met) | 0.04 | Gefitinib, Alisertib, Canertinib | 8.76 |
| rs147597514-T/C(ALDH1L1) | K (Lys) > K (Lys) | 0.03 | Dactolisib, Alisertib, Alvocidib | 8.30 |
| rs72899872-T/C (LRP1B) | A (Ala) > S (Ser) | 0.03 | ABT751, Erlotinib, Navitoclax | 8.51 |
